# Persistence of Structural Distortion and Bulk Band Rashba Splitting in SnTe above Its Ferroelectric Critical Temperature

**DOI:** 10.1021/acs.nanolett.3c03280

**Published:** 2023-12-18

**Authors:** Frédéric Chassot, Aki Pulkkinen, Geoffroy Kremer, Tetiana Zakusylo, Gauthier Krizman, Mahdi Hajlaoui, J. Hugo Dil, Juraj Krempaský, Ján Minár, Gunther Springholz, Claude Monney

**Affiliations:** † Department of Physics and Fribourg Center for Nanomaterials, Université de Fribourg, Fribourg 1700, Switzerland; ‡ New Technologies-Research Center, University of West Bohemia, Plzeň 301 00, Czech Republic; § Institut Jean Lamour, UMR 7198, CNRS-Université de Lorraine, Campus ARTEM, 2 allée André Guinier, BP 50840, Nancy 54011, France; ⊥ Institut für Halbleiter-und Festkörperphysik, Johannes Kepler Universität, Linz 4040, Austria; ¶ Institute of Physics, Ecole Polytechnique Fédérale de Lausanne, Lausanne 1015, Switzerland; # Photon Science Division, Paul Scherrer Institut, Villigen 5232, Switzerland

**Keywords:** ARPES, Rashba effect, ferroelectric transition, SnTe, electronic structure, one-step calculations

## Abstract

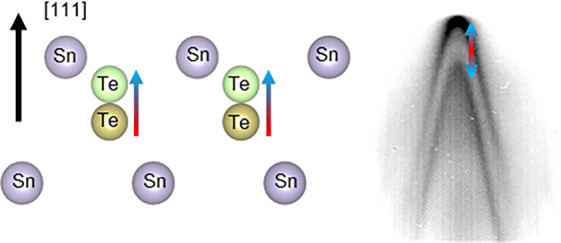

The ferroelectric semiconductor α-SnTe has been regarded as a topological crystalline insulator, and the dispersion of its surface states has been intensively measured with angle-resolved photoemission spectroscopy (ARPES) over the past decade. However, much less attention has been given to the impact of the ferroelectric transition on its electronic structure, and in particular on its bulk states. Here, we investigate the low-energy electronic structure of α-SnTe with ARPES and follow the evolution of the bulk-state Rashba splitting as a function of temperature, across its ferroelectric critical temperature of about *T*_*c*_ ≈ 110 K. Unexpectedly, we observe a persistent band splitting up to room temperature, which is consistent with an order–disorder contribution of local dipoles to the phase transition that requires the presence of fluctuating dipoles above *T*_*c*_. We conclude that no topological surface state can occur under these conditions at the (111) surface of SnTe, at odds with recent literature.

Semiconductors-based spintronics materials are one of the most promising playgrounds for modern applications and technologies.^1,2^ Among these materials, class IV–VI semiconductors are particularly interesting because they can combine semiconductor properties with ferroelectricity. This is due to a spontaneous distortion of the crystalline lattice structure that leads to a macroscopic electric polarization of the material.^3−6^ In addition, the concomitant inversion symmetry breaking induces a momentum-dependent energy splitting in the electronic band structure, i.e., the so-called Rashba effect, which means that these bands are not anymore spin degenerate.^7,8^

In this framework, the electronic band structure of α-GeTe, a ferroelectric Rashba semiconductor with a critical temperature *T*_*c*_ = 670 K,^9^ has been investigated in details. Different surface states, surface resonances, and bulk states have been identified using angle-resolved photoemission spectroscopy (ARPES),^10,11^ and those studies have led to the observation of one of the largest Rashba parameters.^12^ Its potential for application is then particularly large, e.g., with the ability to enhance spin Hall conductivity,^13^ to control the spin-to-charge conversion, to store information in a nonvolatile way,^14−18^ or to manipulate the crystal distortion and thus the ferroelectricity using intense femtosecond pulses.^19^ The isostructural compound SnTe has similar properties to those of GeTe and it shows ferroelectricity typically below 100 K.^20^

For both GeTe and SnTe, the nature of the ferroelectric transition is still subject to debate, even though it has attracted a great deal of attention in the literature. Early studies using neutron diffraction or Raman scattering to reveal the atomic structure and related phonons^9,20,21^ suggested that the transition temperature could strongly depend on the number of Sn (Ge) vacancies and that the transition is of second order.^9,22,23^ Subsequently, this was confirmed by theoretical^24−27^ and experimental studies that demonstrated a phonon softening at *T*_*c*_, indicating a displacive phase transition.^28^ However, extended X-ray absorption fine structure, X-ray scattering measurements, and analysis of the pair distribution function evidenced the persistence of a local rhombohedral lattice distortion above *T*_*c*_, indicating the presence of local ferroelectric dipoles.^29−32^ This conclusion was criticized in another work based on the analysis of pair distribution function,^33,34^ emphasizing that a vivid debate on the ferroelectric phase transition in GeTe and SnTe still remains.

SnTe has been regarded as an outstanding representative of a class of topological crystalline insulators. However, the ferroelectric transition also has considerable effect on its topological properties. Symmetry arguments have been used to claim that in the paraelectric phase this semiconductor has gapless protected surface states.^35^ This was first predicted theoretically,^36,37^ and the existence of linear-dispersive bands attributed to topologically protected surface states was later on confirmed by ARPES for the (100) and (111) surface-plane orientations.^38−43^ However, as shown by Plekhanov et al.,^44^ the topological surface state does not subsist in a ferroelectric state on the (111) surface.

In the present work, we study the low-energy electronic structure of SnTe(111) across its ferroelectric phase transition with ARPES. Taking advantage of our high energy and momentum resolution and also of the unprecedented crystalline quality of our thin films, we reveal multiple states in the first eV below the Fermi level that have not been resolved in the literature so far. Based on one-step model photoemission calculations and photon-energy dependent ARPES measurements, we classify them as surface or bulk states. Most importantly, we systematically characterize the change in the Rashba splitting of bulk states as a function of temperature. We observe clear inconsistencies of the ferroelectric phase transition with a simple mean-field-like transition that can be explained with an order–disorder type contribution to the transition. Finally, we comment on its consequences for the topological properties of the (111) surface of SnTe.

SnTe undergoes a transition from a paraelectric state, with a cubic rocksalt structure with equidistant stackings of Sn and Te layers along the [111] direction (space group Fm3̅m, see Figure 1a), to a ferroelectric state with a rhombohedral structure (space group *R*3*m*, see Figure 1b) at low temperature around 100 K.^20^ In the ferroelectric state, the bulk inversion symmetry is broken by a displacement of the Sn and Te lattice planes against each other, which leads to a nonzero electric dipole between the ionic charges σ^+^ and σ^–^ of the Sn and Te atoms. This induces a Rashba-like splitting in the electronic structure, as can be seen by comparing the bulk DFT band structure in Figure 1d calculated for the paraelectric (blue bands) and ferroelectric (red bands) states. Our objective is to experimentally resolve this splitting and to follow its evolution as a function of temperature in order to characterize the ARPES signatures of the paraelectric-to-ferroelectric phase transition.

**Figure 1:**
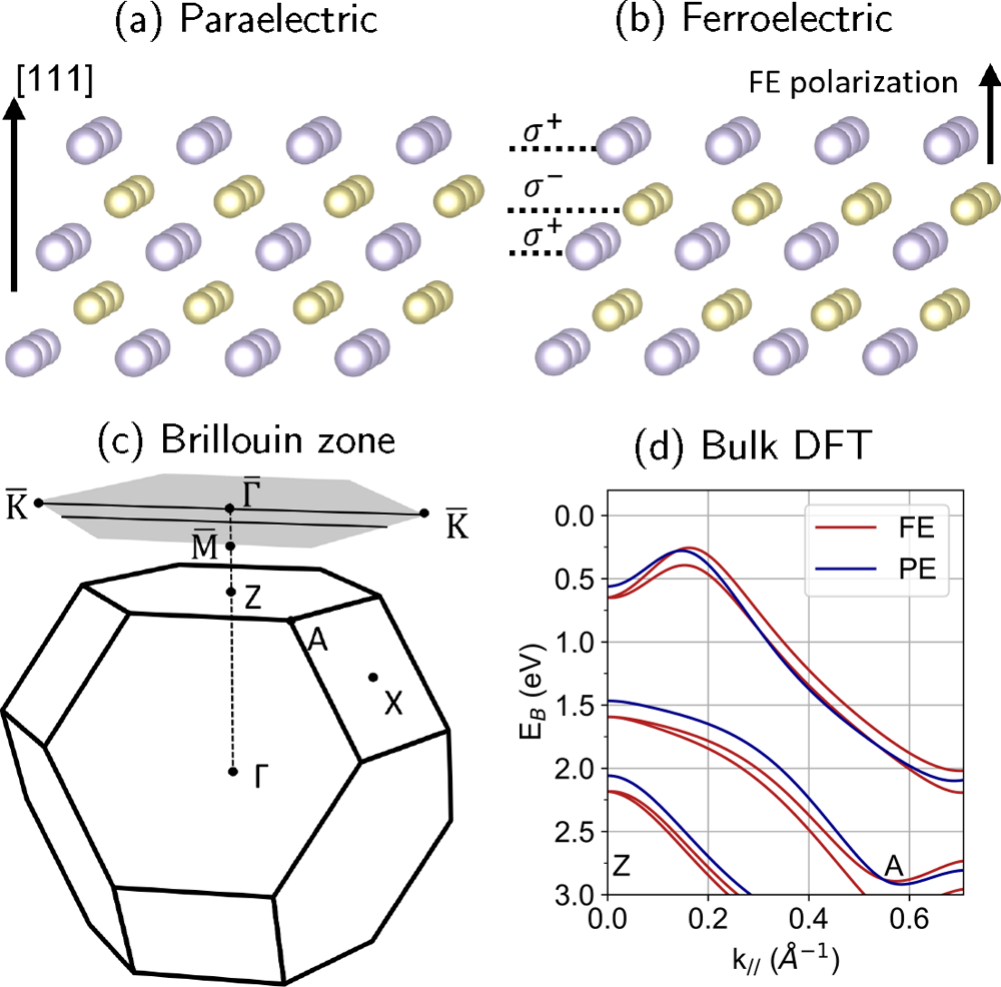
(a) Rocksalt structure of SnTe in the paraelectric state along the [111] crystalline direction. Blue (yellow) dots represent Sn (Te). (b) Rhombohedral structure of SnTe in the ferroelectric state (not to scale). Structures were generated with VESTA.^45^ (c) Bulk Brillouin zone of SnTe and its surface projected plane along the [111] direction. The black lines in the surface projected plane represent the directions of data presented in this work. (d) Bulk DFT band structure between the high symmetry points Z and A. A *k*_⊥_ value of 50% of the Γ*Z* distance has been selected to approximate the reciprocal space plane sampled at a photon energy of 11.2 eV. Bands are plotted for the paraelectric (PE) cubic rocksalt (blue) and ferroelectric (FE) rhombohedral (red) structures.

We have performed ARPES measurements of SnTe(111) along the  high-symmetry direction, which corresponds to the projection of the *AZA* direction on the (111) surface. Photoemission intensity maps obtained at 30 K with two different photon energies *hν* are shown in Figure 2a for *hν* = 11.2 eV and Figure 2b for *hν* = 21.2 eV, respectively. Thanks to the high energy resolution of our experiment and the high quality of our thin films, we distinguish several bands in the low-energy region, where previously only a linear dispersive band was resolved and attributed to a Dirac cone.^39−41^ Near the Fermi level, we identify one surface state (labeled *S*_1_) that appears for both photon energies at higher parallel momenta. At lower momenta, we observe another surface state (*S*_2_) which partially overlaps with a bulk state (*B*_1_) that disperses with photon energy. A more detailed series of photon-energy dependent ARPES measurements using synchrotron radiation is shown in the Supporting Information and corroborates these observations. Similar to the isostructural compound α-GeTe,^10^ we attribute the state *S*_2_ to a surface resonance state.

**Figure 2:**
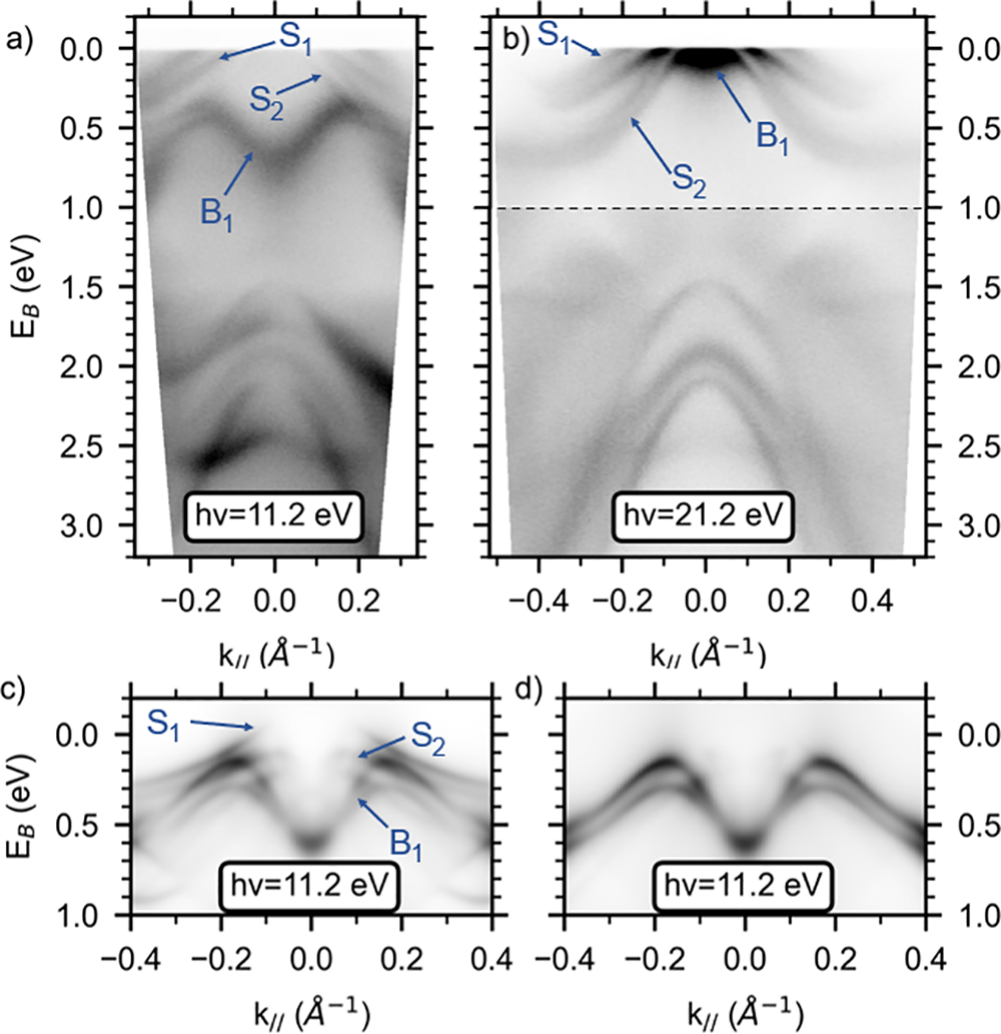
ARPES measurements along the  high-symmetry line at 30 K with a photon energy of (a) 11.2 eV and (b) 21.2 eV. The dashed line in panel (b) represents a change of saturation by a factor of 2. (c) One-step photoemission calculation for a semi-infinite slab geometry in a ferroelectric structure, with a photon energy of 11.2 eV and a Te termination. (d) Same calculation with a transparent barrier.

To support our interpretation of the origin of the bands, we have performed DFT calculations using a semi-infinite slab geometry for the ferroelectric structure. The surface with a Te-termination and short bonds between the first Te and Sn planes gives the best agreement with the experimental data (Supporting Information).

Figure 2c and d show the calculations for a 11.2 eV photon energy with an active and a transparent surface barrier, respectively. The position of the Fermi level in the calculation has been corrected to match with the experiment, and the *k*_⊥_ sampled at 21.2 eV (11.2 eV) has been estimated to be approximately 80% (50%, respectively) of the total Γ*Z* distance. The transparent surface barrier suppresses the surface states and allows us to discriminate the origin of the bands (see Supporting Information for more details about this procedure). The resulting comparison between theory and experiment confirms our attribution of the bands *S*_1,2_ and *B*_1_ to surface and bulk states, respectively.

Having clarified the nature of the low-energy band structure, we focus now on the bulk state *B*_1_ that shows a large energy splitting at low temperature in the ferroelectric phase. We concentrate ourselves now on the data as measured at 11.2 eV photon energy (see Figure 2b), which allows us to disentangle the bulk states from the surface states and to clearly resolve the bulk Rashba splitting. By presenting ARPES measurements as a function of temperature, we address the effect of the ferroelectric to paraelectric transition on the amplitude of the bulk Rashba splitting, which is directly correlated to the ferroelectric distortion.

Figure 3a and b show ARPES spectra taken at 30 K and at 200 K, respectively, with *hν* = 11.2 eV. At 30 K, a clear splitting is observed in all bands, namely the surface ones *S*_1,2_ and the bulk one *B*_1_. At 200 K, the bands become significantly broader due to thermal effects, but a splitting of the surface-related bands *S*_1,2_ is still obvious, in contrast to the bulk band *B*_1_, for which the splitting is no longer clearly resolved. We therefore plot in Figure 3e energy distribution curves (EDCs) integrated over *k*_∥_ ∈ [−0.20, −0.19] Å^–1^ (blue region in panel Figure 3a) for a large temperature range up to room temperature. These EDCs allow us to clearly distinguish the two peaks related to the split bulk band at low temperature (in the ferroelectric phase) and to follow the splitting up to about 160 K. At higher temperature, the two peaks seem to merge together so that it is difficult to directly assess whether the band splitting persists at high temperatures or whether it collapses.

**Figure 3:**
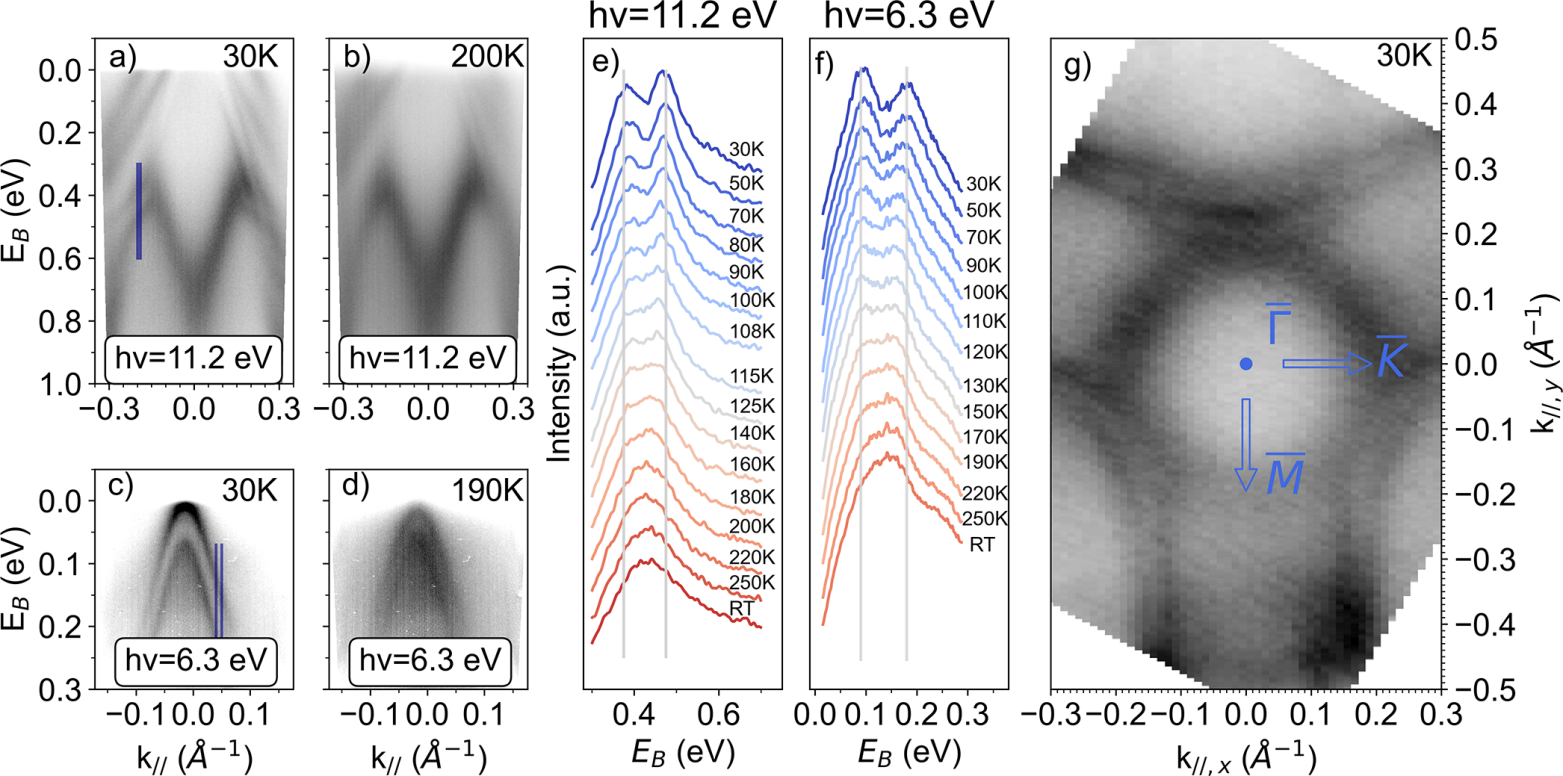
ARPES measurements along the  high-symmetry line (*k*_∥,*y*_ = 0 Å^–1^) with a photon energy of 11.2 eV at (a) 30 K and (b) 200 K. ARPES measurements along the line  shifted in direction to M̅ (*k*_∥,*y*_ = −0.13 Å^–1^) with a photon energy of 6.3 eV at (c) 30 K and (d) 190 K. (e) EDCs as a function of temperature for a photon energy of 11.2 eV, with *k*_∥_ ∈ [−0.20, – 0.19] Å^–1^ (blue region in panel (a)). Light-gray lines are guides to the eye for the reader. (f) EDCs as a function of temperature for a photon energy of 6.3 eV, with *k*_∥_ ∈ [0.04, 0.05] Å^–1^. (g) Constant energy map at binding energy *E*_*B*_ = 0.4 eV, photon energy *hν* = 11.2 eV and at *T* = 30 K.

To answer this question, we have acquired ARPES data using a photon energy of 6.3 eV to take advantage of the higher momentum resolution at lower photon energy. For this purpose and to maximize the effect of the splitting, we oriented the analyzer slit in a plane parallel to , but shifted toward M̅ at *k*_∥,*y*_ = −0.13 Å^–1^ (which allows to see the same bulk band *B*_1_ at a different position in the reciprocal space, see Figure 3g).

The corresponding photoemission intensity maps are shown in Figure 3c and d for temperatures of 30 and 190 K, respectively. We observe two hole-like bands with a clear splitting at low temperature (Figure 3c). One-step model photoemission calculations confirm that these are the same bulk band *B*_1_ (see the calculations with and without a transparent surface barrier in the Supporting Information). Moreover, the band splitting is still visible at 190 K (Figure 3d). We have extracted EDCs in this configuration for *k*_∥_ ∈ [0.04, 0.05] Å^–1^ to follow the reduction of the splitting as a function of temperature (see Figure 3f), which allows us to track the splitting up to 250 K at least.

For a quantitative characterization of the temperature evolution of the bulk states across the ferroelectric transition, we have fitted the EDCs of Figure 3e and f with two Voigt functions (see Supporting Information for more details on the procedure). The variation of the splitting as a function of temperature is plotted in Figure 4. We caution that although we obtained good fits with two Voigt functions for temperatures above 250 K, equally good fits could be obtained with a single broader Voigt function in this temperature range. However, this would lead to an abrupt and nonphysical behavior of the width of the Voigt function around 200 K; therefore, we focus on the scenario with two peaks up to room temperature (a single peak scenario starting above the transition temperature where two peaks are still clearly visible in the EDCs, e.g., at 190 K, gives an absurdly large width. Moreover, this width decreases with increasing temperature, indicating that we are trying to fit with one peak two contributions that are moving closer together.). First of all, we see that the evolution of the band splitting in temperature is the same for the two sets of EDCs, confirming the same mechanism observed with both photon energies. Second, the reduction of the splitting is particularly pronounced below 100 K, but a finite value remains at higher temperatures, up to room temperature, at odds with the expectation for a paraelectric cubic state at high temperature. The evolution of the band splitting was reversible and reproducible across different heating and cooling cycles.

**Figure 4:**
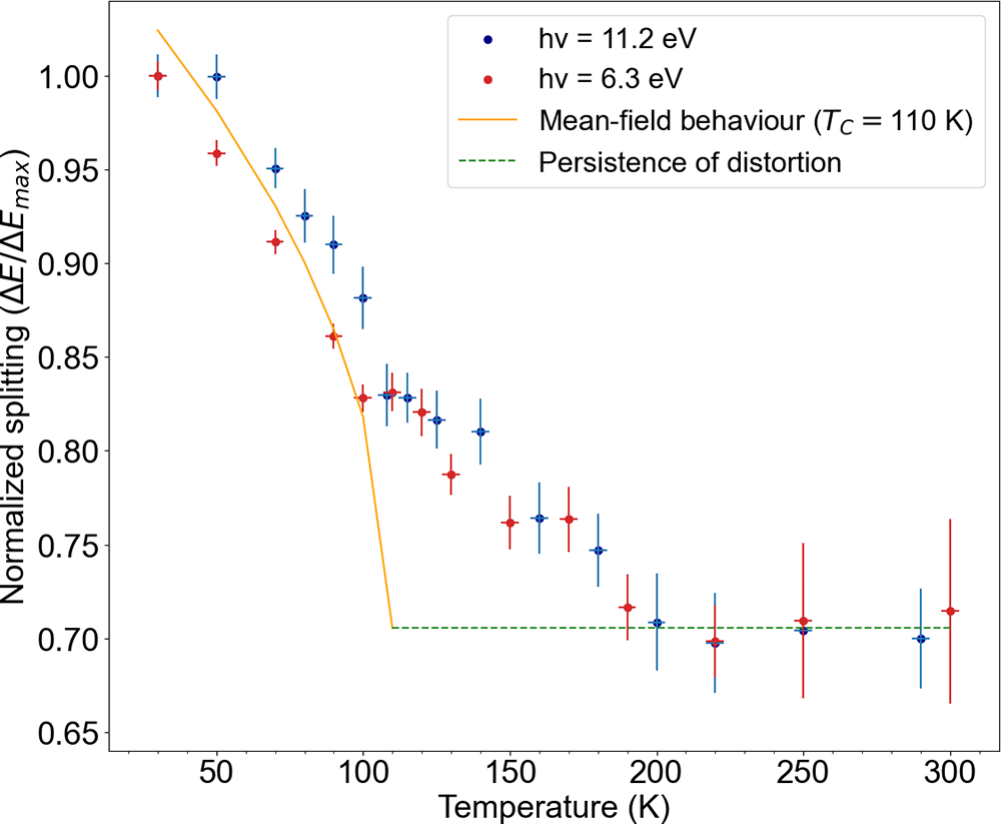
Evolution of the Rashba bulk band splitting as a function of temperature extracted from ARPES data obtained using a photon energy of 11.2 eV (6.3 eV) in blue (red). A mean-field-like second-order phase transition with a *T*_*c*_ = 110 K is added on top (orange curve). However, the nonzero splitting above *T*_*c*_ (dashed green line) indicates the persistence of a structural distortion up to room temperature, in disagreement with a mean-field transition. The error bars are due to temperature measurement precision and the fitting procedure.

Although the origin of the transition (displacive vs order–disorder) remains a matter of debate in the literature, its second-order character is agreed upon.^9,20,21,24−26,28,29,46^ We therefore superimpose on the experimental data in Figure 4 a mean-field-like (orange) curve with a critical temperature of *T*_*c*_ = 110 K and adding a constant offset of Δ*E*/*E*_max_ = 0.7. We stress that within a mean-field-like scenario, one would expect a zero offset at room temperature. The obtained curve agrees well with the low temperature data, but it reveals a rounding of the phase transition above *T*_*c*_. This could be due to strong thermal fluctuations, in agreement with the general increased broadening of the bands observed at and above about 200 K in ARPES (Figure 3b). However, this fails to explain the persistence of a splitting well above *T*_*c*_, which is a clear indication of inversion symmetry breaking inside the crystal, even at high temperature.

This surprising observation is consistent with a contribution from an order–disorder type of phase transition. Whereas for a displacive transition, the onset of the anion/cation displacement appears *only* at *T* ≤ *T*_*c*_ and continuously grows as the temperature decreases, the order–disorder phase transition is based on the ordering below *T*_*c*_ of unit cells that are already distorted above *T*_*c*_, but with random orientation of the anion/cation displacement. As a result, although above *T*_*c*_ the net macroscopic polarization is zero, there are still clusters with a nonzero local polarization extending over a few-unit cells and with alternation of the sign of the polarization from cluster to cluster. These clusters would therefore still have locally a structural distortion and thus give rise to the spin-split bulk bands above *T*_*c*_ as we experimentally observe. This agrees with extended X-ray absorption fine structure and X-ray scattering analysis of the pair distribution function that have revealed the persistence of local lattice distortions, i.e., the presence of local ferroelectric dipoles above *T*_*c*_.^29−32^ We caution though that we cannot rule out another structural mechanism occurring specifically near the surface of SnTe that could cause the persistence of the band splitting at high temperature,^47^ given that ferroelectricity has been observed up to room temperature in the two-dimensional limit of SnTe.^48^

From our data at a photon energy of 11.2 eV (see Figure 2a), we can estimate a minimal value for the Rashba parameter α_*R*_. With the standard relation α_*R*_ = 2*E*_*R*_/*k*_0_, we extract *E*_*R*_ = 0.34 eV and *k*_0_ = 0.19 Å^–1^ at 30 K. The Rashba parameter is then α_*R*_ = 3.58 eV Å. We note that this experimental value is relatively close to the theoretical estimation from DFT in ref (44) (α_*R*_ = 4.4 eV Å), therefore providing an experimental confirmation of the giant Rashba effect in SnTe.

Given our discovery of the persistence of a structural distortion in SnTe at higher temperatures, an open question is what is its impact on the topological surface states? Symmetry arguments have been used to derive a nonzero mirror Chern number on the (001), (111), and (110) surfaces of the rocksalt paraelectric structure and therefore the presence of Dirac cones in the ARPES spectra.^35^ Such considerations were supported by earlier theoretical^36,40^ and experimental studies on the (001) surface.^38^ As for the (111) orientation, Plekhanov et al. showed that in a rhombohedral distorted structure there are no gapless topological surface states near the Fermi level. In that respect, static ARPES studies have claimed to have measured a topological surface state at Γ̅.^39−41^ However, the highly p-type character of SnTe precludes the direct observation of the Dirac cone by ARPES. Our results provide a new perspective to these findings by resolving more bands, namely, two surfaces states instead of one, and with an unprecedented resolution. By looking at our ARPES measurements, we identify the *S*_1_ and *S*_2_ surface states as the candidate for the linear dispersion in the occupied states that has been interpreted as a topological surface state in previous studies. In light of the study of Plekhanov and co-workers,^44^ our observation of the persistence of local rhombohedral lattice distortions above *T*_*c*_ therefore excludes the possible existence of topological surface states at high temperature on the (111) surface, at odds with recent time-resolved ARPES studies.^49^ Our new results therefore require a reassessment of these observations.

We have characterized the band structure of SnTe(111) using high-energy resolution ARPES measurements with unprecedented quality. Combined with state-of-the-art photoemission calculations with and without a surface barrier, our ARPES study at selected photon energies enabled us to differentiate surface and bulk states. The presence of bulk-split bands has been directly connected to the inversion symmetry breaking. We also studied the evolution of this splitting as a function of temperature to characterize the ferroelectric transition. This study demonstrated inconsistencies with a displacive mean-field like transition, revealing a rounding of the phase transition and a splitting persisting above *T*_*c*_, at least up to 250 K. This observation is consistent with an order–disorder type phase transition, in agreement with findings from other studies using local probes.^29,30,47^ Above the critical temperature, fluctuations of the polarization vector from one cluster to another imply that a structural distortion remains and explain the persistence of the band splitting at high temperature. We propose that the possible persistence of ferroelectricity at high temperature in the near-surface region could be tested with spin-resolved and microfocus ARPES measurements by looking for the existence of a finite spin polarization, as well as by evidencing circular dichroism in ARPES.^50,51^ Finally, the persistence of rhombohedral distortions above the critical temperature requires a reassessment of the topological nature of the SnTe(111) surface since it has been shown in the literature^44^ and confirmed by our DFT calculation that the break of symmetry destroys the topological surface state along the (111) direction.

## Supporting Information Available

Details on the samples growth and characterization; methods used to perform the ARPES measurements and the calculations; photon-energy dependent ARPES measurements; one-step photoemission calculations for the 6.3 eV ARPES data; DFT calculations for different surface terminations; fitting of the Rashba splitting (PDF)
