# Supplementary material for: Persistence of Structural Distortion and Bulk Band Rashba Splitting in SnTe above Its Ferroelectric Critical Temperature
Source: Nano Lett. 2023 Dec 18;24(1):82–8. doi: 10.1021/acs.nanolett.3c03280 (PMC10786156; doi:10.1021/acs.nanolett.3c03280)
Supplement: Supplementary file 1 — nl3c03280_si_001.pdf [file nl3c03280_si_001.pdf]

Supporting Information for "Persistence of structural distortion and bulk band Rashba splitting in SnTe above its ferroelectric critical temperature"

Frédéric Chassot,[*,†] Aki Pulkkinen,[†,‡] Geoffroy Kremer,[†,¶] Tetiana Zakusylo,[§] Gauthier Krizman,[§] Mahdi Hajlaoui,[§] J. Hugo Dil,[‖,⊥] Juraj Krempaský,[⊥] Ján Minár,[‡] Gunther Springholz,[§] and Claude Monney[*,†]

†*Department of Physics and Fribourg Center for Nanomaterials, Université de Fribourg, Fribourg, 1700, Switzerland*

‡*New Technologies-Research Center, University of West Bohemia, Plzeň, 301 00, Czech Republic*

¶*Institut Jean Lamour, UMR 7198, CNRS-Université de Lorraine, Campus ARTEM, 2 allée André Guinier, BP 50840, Nancy, 54011, France*

§*Institut für Halbleiter-und Festkörperphysik, Johannes Kepler Universität, Linz, 4040, Austria*

‖*Institute of Physics, Ecole Polytechnique Fédérale de Lausanne, Lausanne, 1015, Switzerland*

⊥*Photon Science Division, Paul Scherrer Institut, Villigen, 5232, Switzerland*

E-mail: frederic.chassot@unifr.ch; claude.monney@unifr.ch

Methods

Sample growth

Epitaxial SnTe(111) films of 2 µm thickness were grown by molecular beam epitaxy on BaF_2 substrates under ultra-high vacuum (UHV) conditions at a substrate temperature of 350° C and a compound effusion cell. During growth, the SnTe(111) surface exhibits a perfect two-dimensional reflection high-energy electron diffraction pattern revealing a perfect 2D growth mode. After growth, the samples were transferred to the ARPES setup without breaking UHV conditions using a battery operated vacuum suitcase having a pressure of better than 10^{-10} mbar. It is noted, that due to the high density of native Sn vacancies, SnTe intrinsically exhibits a high p-type carrier concentration of typically 2 x 10^{20} cm^{-3}. For this reason the Fermi level is always inside the topmost valence band. At room temperature, the lattice parameter of the SnTe layers was determined to be $a = 6.325$ Å(rhombohedral lattice parameter of 4.472 Å), which is in good agreement with literature values.[1]

X-ray diffraction : The SnTe epilayers were characterized by high resolution x-ray diffraction using a Seifert 3003 PTS x-ray diffractometer equipped with a channel cut Ge (220) monochromator set for a wavelength of 1.5406 Å. As shown by Fig. S1a in the symmetric $\omega/2\theta$ radial scan of our 2 µm thick SnTe layer on BaF_2 (111) substrate, exclusively sharp (hhh) Bragg peaks from the SnTe layer and the substrate appear, as indicated. Accordingly, the SnTe layer is perfectly single crystalline and oriented with the (111) lattice planes parallel to the surface. From the peak positions, the SnTe lattice parameter was derived at room temperature as $a = 6.325$ Å, in good agreement with literature values.[1–4] The corresponding rhombohedral lattice parameter is 4.472 Å$(= a/\sqrt{2})$. The high quality of our SnTe films is further evidenced by the x-ray reciprocal space map (RSM) shown in Fig. S1b for a 50 nm SnTe thin film on BaF_2 (111). Evidently, the peak width of the SnTe layer is as narrow of that of the BaF_2 substrate. In addition, pronounced satellite peaks in the Q_z direction appear arising from the finite thickness of the SnTe layer, which is another

indication of the high sample quality.

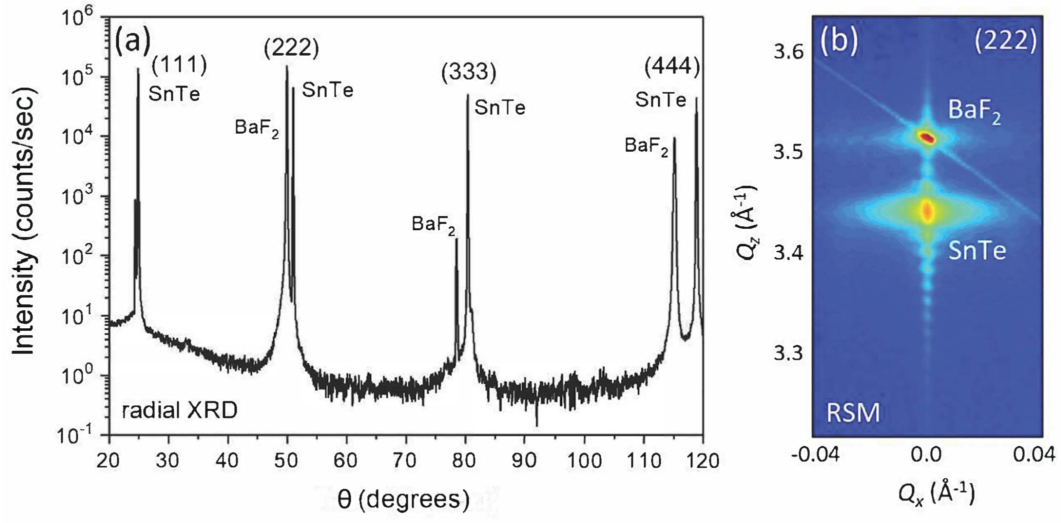

Figure S1: X-ray diffraction characterization of SnTe epitaxial films grown on BaF_2 (111) substrates. (a) Radial XRD scan of a 2 µm film along the [111] surface direction. (b) x-ray reciprocal space map around the (222) Bragg reflection for a SnTe thin film with 50 nm thickness. The data set evidences the high structural quality of the samples.

ARPES measurements

Temperature-dependent angle-resolved photoemission spectroscopy (ARPES) investigations were carried out using a Scienta DA30 photoelectron analyzer with a base pressure better than 3×10^{-11} mbar. Photons sources are monochromatized He_I (and Xe) radiation with $h\nu = 21.22$ eV ($h\nu = 11.2$ eV) and a high energy-resolution laser based on a commercial setup (Harmonix, APE GmbH) generating 6.3 eV photons using harmonic generation from the output of an optical parametric oscillator pumped by a Paladin laser (Coherent, inc.) at 80 MHz. The total energy resolution was about 10 meV and cooling of the sample was carried out at rates <2 K/min to avoid thermal stress. Each measurement was preceded by a break of at least 15 min, to ensure thermalisation. Accordingly, the error on the absolute sample temperature was estimated to be well below 5 K. The photon-energy dependent ARPES measurements were performed at 30 K at the URANOS beamline of the SOLARIS synchrotron in Krakow using a Scienta DA30L photoelectron analyzer.

Photoemission calculations

The ARPES calculations were performed using the one-step model of photoemission implemented in the multiple scattering Green's function code SPRKKR.[5,6] The bulk electronic structure was calculated within the atomic sphere approximation with angular momentum expansion up to $l_{\max} = 3$ with lattice parameter $a = 4.4547$ Å, rhombohedral distortion angle 59.9 degrees, and $z_{\mathrm{Te}} = 0.52$. To investigate the origin of the spectral features, we have compared one-step model calculations with the Rundgren-Malmström model surface barrier and a transparent barrier, which allows us to evaluate contributions from surface-related bands.[7]

In the layer-KKR formalism of the one-step model of photoemission,[8,9] the crystal structure is divided into layers whose transmission and reflection factors characterize the photocurrent attenuation inside the crystal. In addition to the atom layers, the Rundgren-Malmström[10] type surface barrier connecting the inner potential of the crystal to the vacuum level is also treated as a layer with transmission and reflection factors. The surface barrier layer reflection factor is set to zero in the transparent barrier calculations.

Density functional theory calculations

Density functional theory (DFT) band structure calculations with spin-orbit coupling were performed using the Vienna ab-initio software package (VASP).[11–15] The SnTe(111) surface was modeled as a repeated slab geometry of 72 atom layers and 24 Å of vacuum between the adjacent slabs. Exchange and correlation (xc) effects were treated at the level of the generalized gradient approximation using the PBE xc-functional.[16] The kinetic energy cutoff was set to 250 eV, and a 9×9×1 k-point mesh was used for the Brillouin zone sampling in the slab calculations.

Photon-energy dependent ARPES measurements

Figure S2 shows photon-energy dependent ARPES measurements taken along the $\overline{K\Gamma K}$ high-symmetry direction at 30 K with photon energy ranging from 13 to 22 eV. These data allows to follow the $k_\perp$ dispersion of the different bands identified in the main text. Although the intensity of the surface states S_1 is lower than in the main text (probably due to a slightly degraded surface quality), we observe that the states S_1 and S_2 do not disperse as function of photon energy, contrarily the state B_1. These data confirms our identification of S_1 and S_2 as surface states and B_1 as a bulk state.

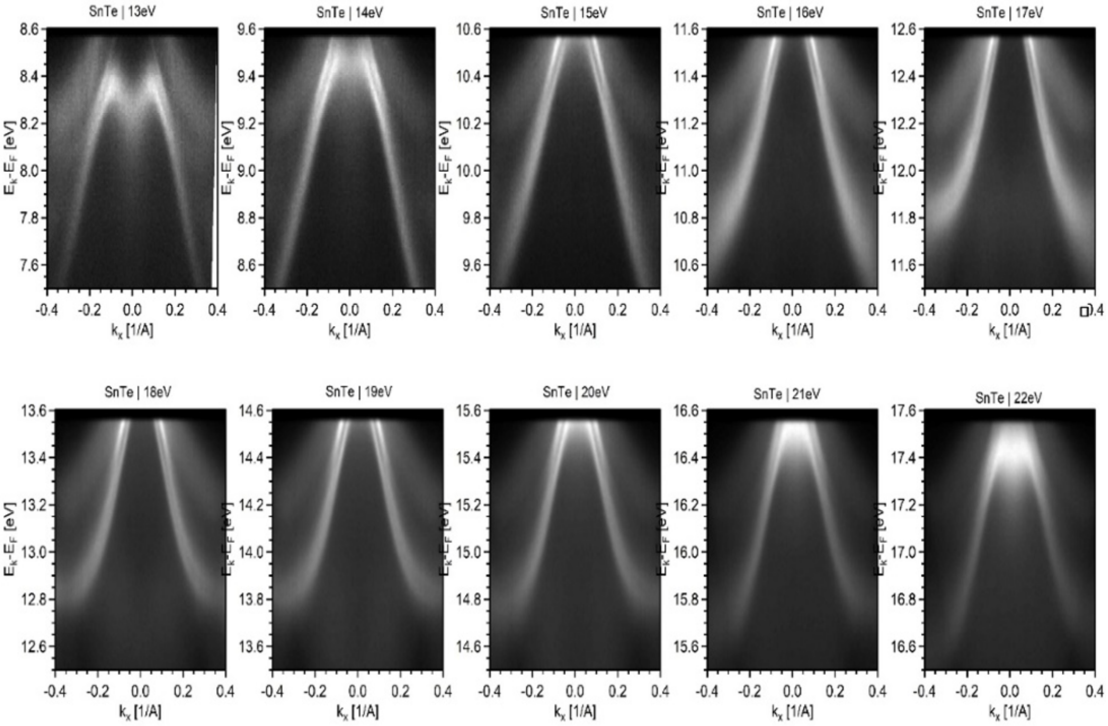

Figure S2: Photon-energy dependent ARPES measurements along the $\overline{K\Gamma K}$ high-symmetry direction at 30 K with a photon energy ranging from 13 to 22 eV as indicated.

Photoemission calculation for the 6.3 eV ARPES data

Figures S3a and S3b displays the one-step photoemission calculation with and without, respectively, the surface barrier at a photon energy of 6.3 eV along the same wave vector

direction as used in the experiment. Despite slight differences with the experimental data in Fig. 3c of the main text (e.g., a very weak surface state in the calculations that seems to be hidden in the background of the measurement), the qualitative comparison is good and proves that the investigated band is a bulk one.

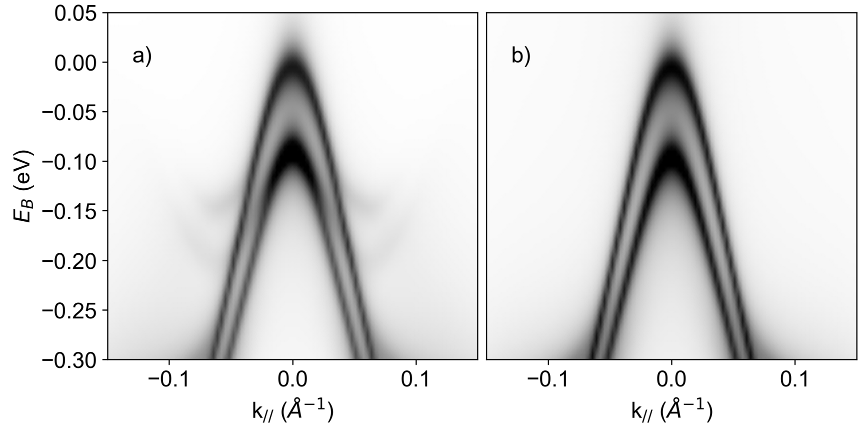

Figure S3: One-step photoemission calculations for a semi-infinite slab geometry in a ferroelectric state, with a photon energy of 6.3 eV and a Te termination, along a line parallel to $\overline{K\Gamma K}$ shifted towards $\overline{M}$ at $k_{\parallel,y} = -0.13$ Å^{-1} without (a) and with (b) a transparent surface barrier.

DFT calculations for different surface terminations

Figure S4 displays the DFT calculation of SnTe(111) in its ferroelectric state for four different terminations, namely the Te or Sn terminated surfaces, and for each case with a short or a long distance between the first two atomic planes (called short and long configurations thereafter). The bulk projected bands are integrated in the grey area and the contribution of the first surface layer is highlighted in yellow or blue dots, for the long and short configurations, respectively. As such, we expect to visualize the surface state S_1 (by analogy with GeTe, S_2 being interpreted as a surface resonance state having a deeper origin in the crystal[17]). The comparison with the experimental data (see Fig. 2 of the main text) confirms our interpretation of the origin of the bands and clearly proves that our samples are Te terminated. The short distance configuration seems more likely, because of the presence of surface states with higher binding energies and because its work function (5.05 eV versus

5.45 eV for the long configuration) is closer to the experimental value (4.65 eV).

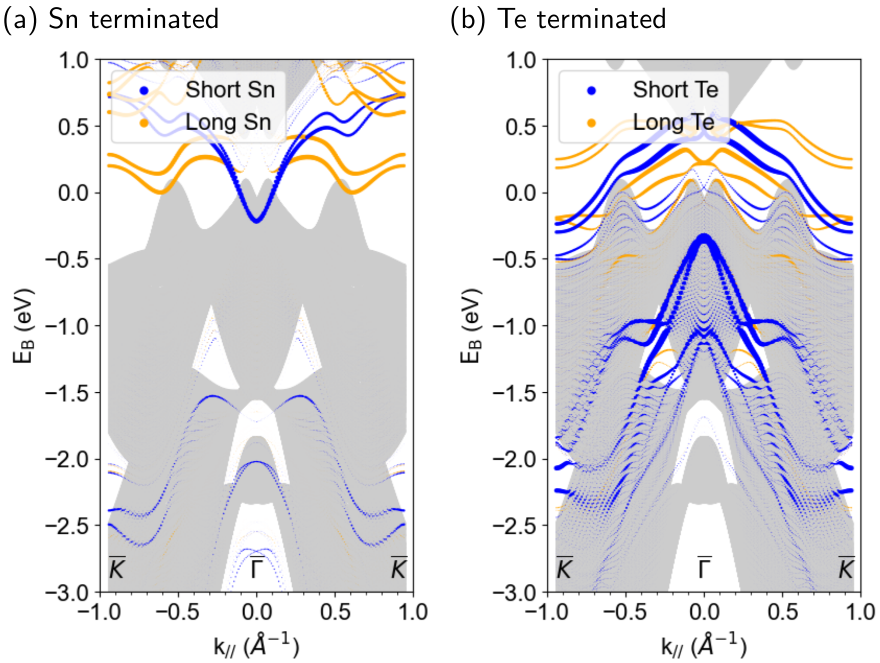

Figure S4: (a) DFT calculation of SnTe(111) made with VASP with a (a) Sn surface termination and (b) a Te surface termination. The shaded area is the bulk projected bands and the size of the blue and yellow markers represents the contribution of the top surface atoms to the character of the bands for the long and short configurations, respectively (see text).

Fitting of the Rashba splitting

Figure S5 illustrates how the fits of the energy distributions curves (EDCs) were done in order to extract the Rashba splitting as a function of temperature. The combination of two Voigt functions with a linear background was used to fit the EDCs on a limited energy range, using the Levenberg-Marquardt algorithm of the lmfit software.[18] No other constraints were imposed on the fit, apart from a manually chosen first guess. The splitting is determined as the distance between the centers of the two Voigt functions.

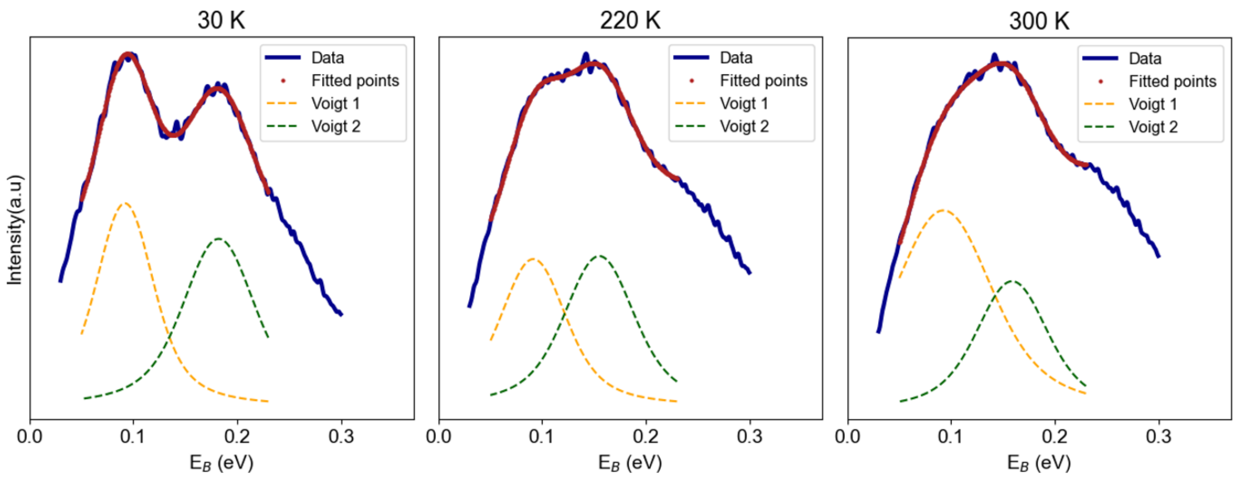

Figure S5: Exemplary fits (red) the EDCs (blue) at a photon energy of 6.3 eV to extract the Rashba splitting as a function of temperature. The orange and the green curves represents the two contributions of a Voigt function.

References

(1) Iizumi, M.; Hamaguchi, Y.; F. Komatsubara, K.; Kato, Y. Phase Transition in SnTe with Low Carrier Concentration. *J. Phys. Soc. Jpn.* **1975**, *38*, 443–449.

(2) Qu, Q.; Liu, B.; Liu, H.; Liang, J.; Wang, J.; Pan, D.; Keong Sou, I. Role of topological surface states and mirror symmetry in topological crystalline insulator SnTe as an efficient electrocatalyst. *Nanoscale* **2021**, *13*, 18160–18172.

(3) Schreyeck, S.; Brunner, K.; Molenkamp, L. W.; Karczewski, G.; Schmitt, M.; Sessi, P.; Vogt, M.; Wilfert, S.; Odobesko, A. B.; Bode, M. Breaking crystalline symmetry of epitaxial SnTe films by strain. *Physical Review Materials* **2019**, *3*, 024203.

(4) Díaz, B.; Henrique Oliveira Rappl, P.; Abramof, E. Molecular beam epitaxial growth of EuTe/SnTe strained superlattices. *Journal of Crystal Growth* **2007**, *308*, 218–222.

(5) Ebert, H.; Ködderitzsch, D.; Minár, J. Calculating condensed matter properties using the KKR-Green's function method—recent developments and applications. *Reports on Progress in Physics* **2011**, *74*, 096501.

(6) Braun, J.; Minár, J.; Ebert, H. Correlation, temperature and disorder: Recent developments in the one-step description of angle-resolved photoemission. *Physics Reports* **2018**, *740*, 1–34.

(7) Rundgren, J.; Malmstrom, G. Transmission and reflection of low-energy electrons at the surface barrier of a metal. *Journal of Physics C: Solid State Physics* **1977**, *10*, 4671.

(8) Pendry, J. Theory of photoemission. *Surface Science* **1976**, *57*, 2.

(9) Braun, J.; Minar, J.; Ebert, H. Correlation, temperature and disorder: Recent developments in the one-step description of angle-resolved photoemission. *Physics Reports* **2018**, *740*, 1.

(10) Malmstroem, G.; J. Rundgren, J. A program for calculation of the reflection and transmission of electrons through a surface potential barrier. *Computer Physics Communications* **1980**, *19*, 2.

(11) Kresse, G.; Hafner, J. Ab initio molecular dynamics for liquid metals. *Phys. Rev. B* **1993**, *47*, 558–561.

(12) Kresse, G.; Hafner, J. Ab initio molecular-dynamics simulation of the liquid-metal–amorphous-semiconductor transition in germanium. *Phys. Rev. B* **1994**, *49*, 14251–14269.

(13) Kresse, G.; Furthmüller, J. Efficiency of ab-initio total energy calculations for metals and semiconductors using a plane-wave basis set. *Computational Materials Science* **1996**, *6*, 15–50.

(14) Kresse, G.; Furthmüller, J. Efficient iterative schemes for ab initio total-energy calculations using a plane-wave basis set. *Phys. Rev. B* **1996**, *54*, 11169–11186.

(15) Kresse, G.; Joubert, D. From ultrasoft pseudopotentials to the projector augmented-wave method. *Phys. Rev. B* **1999**, *59*, 1758–1775.

(16) Perdew, J. P.; Burke, K.; Ernzerhof, M. Generalized Gradient Approximation Made Simple. *Phys. Rev. Lett.* **1996**, *77*, 3865–3868.

(17) Krempaský, J. et al. Disentangling bulk and surface Rashba effects in ferroelectric α-GeTe. *Physical Review B* **2016**, *94*, 205111.

(18) Newville, M.; Stensitzki, T.; Allen, D. B.; Ingargiola, A. LMFIT: Non-Linear Least-Square Minimization and Curve-Fitting for Python. 2014; `https://doi.org/10.5281/zenodo.11813`.
